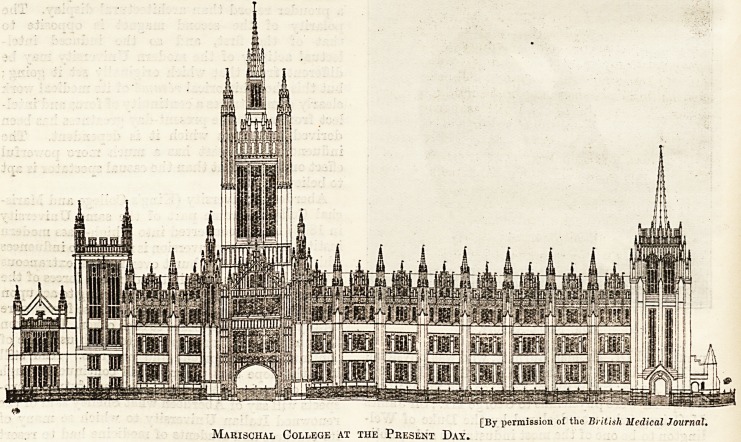# The Celebrations of the Quatro-Centenary of Aberdeen

**Published:** 1906-09-29

**Authors:** 


### THE CELEBRATIONS OF THE QUATRO-CENTENARY OF ABERDEEN.

### MEDICAL INTERESTS AND MEDICAL MEN: AN HISTORICAL RETROSPECT.

### THE PRE-UNIVERSITY ERA IN SCOTLAND.

THIS week the University of Aberdeen celebrates its four hundredth anniversary. Instinctively one thinks of the storied past and wonders if it is possible to trace by what devious ways the life which has endured for four hundred years has been transfused into the present. It is the department of medical and surgical progress that chiefly attracts readers of THE HOSPITAL and which most merits our attention, because it so readily and simply presents a comprehensive view of the place held by Scottish science among other nations and enables us to grasp the great importance of this week's rejoicings in the Granite City. It may be found at times that England was ahead of Scotland in the healing art. The reason was purely political, and, given a period of civil quiet, Scotland evinced both enthusiasm and originality in her efforts to advance both medicine and surgery. The fourteenth century was eventful for England scientifically—for Scotland politically. From 1450 onwards the conditioning circumstances of both countries were more assimilated, if not identical, and under such equality of conditions Scotland shot rapidly ahead and completely outdistanced England. It had already done so in the public medicine department. During times of plague and pestilence the Church in England was the only guardian of the common weal, and discharged its duties in rather a fatalistic spirit, meeting disease rather in the mood of passive acceptance than of scientific and sanitary warfare. In Scotland as early as 1000 A.D. epidemics were guarded, markets supervised, and friendly aid voted to the indigent after the most modern style.

In the north, long before the University era, Scotland was educationally active, and proved herself in many respects quite abreast of her more highly favoured Enropean neighbours. In spite of the political turmoil which agitated Scotland in the early part of the fourteenth century, and probably due to that cause, Scotland even then had eminent medical practitioners and surgeons, but no medical institution. At that period of her history her sons had to fight for their political freedom, to resist what they considered tyranny and oppression, and to oppose the "proud usurpers." It is not greatly to their discredit if the Scot at home had not much time to think of "doctoring."

Originally medicine was mixed up with other studies, and in many cases was looked on as a subject of liberal culture. The purely professional man seems to have had his origin first at Court, next as a baronial adviser, and finally came to be at the service of both King and commoner. And many Scottish names shine in these early years of Scotland's national history among the learned and philosophical doctors of other lands.

THE MARISCHAL COLLEGE IN THE NINETEENTH CENTURY WITH THE MCGRIGOR MEMORIAL OBELISK.

### THE FOUNDING OF THE UNIVERSITIES.

After 1350 political conditions rendered it possible for Scottish students to find both comfort and culture at Oxford, and the zeal of the Church, conjoined with the enthusiasm of the great Scottish municipalities, were the means of bringing into existence those famous seats of learning and progress the Scottish Universities. The first to be founded was St. Andrews, in 1411; then Glasgow, in 1450; then Aberdeen University (King's College), which was founded in 1496—the gift to the North of Scotland of its greatest and best Bishop, William Elphinstone. The foundation-stone was laid in 1500, and the educational work began in 1505—a very noteworthy era in the history of Scottish medicine. Marischal College was founded in 1593. These institutions were monastic in their discipline, though secular and liberal to a degree in their curriculum. In St. Andrews and Glasgow Universities the faculty of medicine was held to be a section of the theological faculty. It was different, however, with Aberdeen, which from the first regarded medicine and surgery to be unique enough to have a special professor and to constitute a special faculty.

THE FIRST PROFESSOR OF MEDICINE IN GREAT BRITAIN.

Thus it is we may take James Cumyne, the first Professor of Medicine in Aberdeen University, as the " Avatar " of the purely professional physician and surgeon, and indeed for Britain generally. This professor was paid for his work at the rate of ten " merks " yearly—paid mostly in victuals and grain. To eke out this scanty livelihood he was granted, on the plea of poverty, the right of salmon-fishing in the Don. He was a highly cultured man, and had assisted the Scottish historian, Hector Boece (who was the first Principal of Aberdeen University), in composing his Scottish History. Cumyne devoted himself principally to " those flowers of choice observations in natural history " which enrich that ancient work on Scottish history.

ROYAL FAVOUR POPULARISES MEDICINE.

Medicine was becoming popular in Scotland about the time when Aberdeen University was founded. The doctors of Scotland at that period had all been trained abroad. No medical school or corporation existed in Scotland until within a few years of the death of King James IV. Scottish kings had practised medicine before James IV.'s time, and many royal personages took interest in medical science. Alfred the Great made the first translation of medical works into the Saxon tongue. Edward the Confessor, who founded Westminster Abbey (980 A.D.), deemed his touch curative—a virtue popularly esteemed to have been hereditarily transmitted to a long lint of royal successors. Charles II. and George III. were also deeply interested in all scientific work, encouraged it, and made it popular. Hence, to some extent, the great results that were then achieved. Royal interest popularises and stimulates public effort. James IV. erected laboratories at Stirling and Edinburgh, with a well-trained foreigner—John the Leeche—in charge of them. It was this John who devoted so much research to the subject of flight and ballooning —a study which has recently been revived. He had conceived the notion that huge wings could enable him to break a fall when jumping from a height—the notion of the parachute—and thought he had succeeded in solving the problem. He attempted to fly " off the Castell walls of Strivelling (Stirling) with ane pair of winges of fedderis fessenit apoun him," with the result that he " shortly fell to the ground and brak his thee bane " (thigh bone). He ascribed his failure to the fact that he had used the wrong materials, for, he said, " thair was sum hen fedderis in the wingis, quhilk yarnit and covet the mydding and not the skyis." The hens' feathers were unsuitable for flight simply because the bird preferred searching for food on a dunghill. This king actually practised medicine. On one occasion he purchased some surgical instruments—" three compasses, ain hammer and a turcase to tak out teeth "—and proceeded to use them upon such of his lieges as could be induced to submit to the operation for a sum of money.

[By permission of the *British Medical Journal*.

MARISCHAL COLLEGE IN THE EIGHTEENTH CENTURY.

It was about this period that the trammels of traditional medicine and the worship of ancient authorities began to give way before the researches of anatomical science. Mediævalism died hard, if dead it be. A very hard task it was for medicine and surgery to turn their backs on the Arab—on Toledo, Bologna, Montpelier, and Salerno.

Medicine was in this way becoming popular when Aberdeen University was founded. The establishment in the Chair of Medicine in the University of Aberdeen shows this. Shortly afterwards the grant of a charter to the members of the Surgeons' Corporation in Edinburgh gives further proof of the awakening. This is the first indication of that true scientific spirit which characterises the development of medical science in Scotland. It is a proof on the part of the municipal authorities of a growing appreciation of better methods and an endeavour to keep abreast of the advances then being made in medical science by other countries of Europe.

### Medical Advance Suspended.

In Scotland this awakening was, unfortunately, not long maintained. Revolution, war, political turmoil intervened to suspend the work of the Universities in the direction of medical progress for nearly three hundred years. The Scottish Universities about the middle of the sixteenth century might, so far as medical matters were concerned, have been asleep; and at Aberdeen, which started so well as a medical centre, medical teaching soon fell into a state of decrepitude and decay. Public interest in medical studies fell off to such a degree that in one University the authorities passed the following minute: "Anent the profession of medicine the 'visitation' finds that profession is not necessary for the College in all time coming, but withal finds it just that the present holder, who is alreadie in that profession, continue in the same during his time."

### "Aberdeen Mediciners" and their Work.

Cumyne died in 1522, and was succeeded in the chair by Robert Gray, who held the appointment for more than forty years, and in turn gave place, in 1566, to a very famous "Doctoure in Medicine," Maister Gilbert Skeyne, whose name is known to Scottish literature as having published "Ane Breve Description of the Pest (Plague), quharin the causis, signis, and some speciall preservatioun and cure thairof are continent set furth"—one of the first medical works printed in Edinburgh by Robert Lekpreok in the year 1568. After Skeyne, Patrick Dun was "Mediciner" in Aberdeen. He was chiefly distinguished by leaving King's College to become the first lay Principal of Marischal College.

### The First Medical Graduate in this Country.

Aberdeen University did not grant degrees in medicine until 1654, when, on May 15 of that year, the degree of M.D. was conferred on an American citizen—James Glover—a native of London, who had taken his degree of B.A. in Harvard University in the year 1650. This American graduate of Harvard became the first medical graduate of Aberdeen University, and consequently the first medical man to obtain an M.D. degree in Scotland. An extraordinary fact of these early days of Aberdeen graduation is that no Scotchman (with one exception) graduated in Aberdeen for the first seven years after the introduction of the Aberdeen degree. The graduates were either Englishmen or Americans, with the exception of Robert Thorner, an Aberdonian, who became an M.D. of Aberdeen in 1665. Among the other notabilities connected with this university more or less directly may be mentioned Dr. Robert Stroloch, Dr. Moresin, an intimate friend of Lord Bacon; James Cargill, who left many benefactions to Marischal College.

### The Aberdeen Scot Abroad.

In the early part of the seventeenth century the Scottish physician was a scholar and philosopher. Scarcely a Continental school existed without its Scotch Professor. William Barclay, of Aberdeen, the Tobacco Doctor, was teaching botany at Oxford, holding that "Tobacco was the mercure of vegetals, and mercure the tobacco of minerals." Barclay (died about 1630) wrote a work, "Caller hoë, or the Nymph of Aberdeen resuscitat," a de-

Duncan Liddle, M.D.

scription of a mineral water cure once greatly in vogue in the Aberdeen district, which in his time had fallen into disuse. In the year 1604 another Aberdonian, Dr. Gilbert Jack, was Professor of Philosophy at the University of Leyden (founded 1575), and then at the height of its glory. Duncan Liddle, another Aberdonian, was the life and soul of Helmstadt, first as a teacher of mathematics and then as Professor of Physics. A memorial brass tablet in the town church of Aberdeen is dedicated "To the Eternal Memory of Duncan Liddle, Doctor of Medicine," and describes him as "eminent in medicine and all philosophy and mathematics." He is represented in the garb of the seventeenth century literary doctor, with a quill pen in his hand and an open book before him.

It is a question whether the town of Aberdeen made these men great or whether it was their fame and reputation that glorified the University Town. The townsmen had to seek elsewhere for medical knowledge, which at this period of her history was denied them at home. Dr. Arthur Johnston, for example, had to go to Padua and Mantua for his medical learning. He became the very highest type of the learned physician of that period, and afterwards was physician to Charles I. of England. He was contemporary with Milton, wrote Latin poetry and Latin prescriptions. The "Dunciad" mentions him in the couplet referring to Benson, the publisher of that day—

> On two unequal crutches propt he came,
> Milton's on this, on that one Johnstone's name.

Aberdeen was the birthplace of William Davisson, who introduced the teaching of chemistry into

France in 1647. His writings and experiments made his name famous throughout all Europe.

PUBLIC DISSATISFACTION WITH MEDICAL TEACHING.

It is small wonder that the public commenced to grumble that no fruit or benefit of any kind was accruing from the medical lectures given in the Scotch

DR. ARTHUR JOHNSTONE.

Universities, and to complain that they were being gulled by " meer cifers and shaddous." Edinburgh municipal enterprise in granting the surgeons permission to dissect led one notable Aberdeen " mediciner "—Dr. Willian Gordon—to a strenuous effort to obtain similar privileges for Aberdeen teachers as had been granted to the surgeons of Edinburgh; and happily in this endeavour he was successful in the year 1636, nearly a century and a half behind Edinburgh. Dr. Andrew Moore followed (1672), and then Dr. Patrick Urquhart occupied the chair till 1725.

THE AGE OF THE GREGORYS.

He was succeeded by Dr. James Gregory, a scion of a remarkable family. The Rev. John Gregory had two sons—David, who was tried by the Presbyter for keeping a barometer, and James, who studied at Padua and invented the Gregorian reflecting telescope. David's family devoted themselves to mathematics, and became professors either of mathematics or astronomy or modern history in the Universities of Edinburgh, St. Andrews, or Oxford. The other line devoted its energies to medicine and its allied sciences, of whom the first was the Professor of Medicine in Aberdeen, and father-in-law of Jamesone, the Scottish Vandyke. He lived between the years of 1674 and 1731, having been appointed Professor of Medicine at King's College, Aberdeen, in 1725. He was a cousin of Rob Roy. It was this Dr. Gregory who wanted to take Rob Roy's son, James Roy (afterwards of Edinburgh), to Aberdeen University " to make a man of him." He was succeeded by his son, Dr. James Gregory (secundus), who was in turn succeeded by his more celebrated brother John in 1755. John had studied at Leyden and was well acquainted with medical progress, and tried to make the Aberdeen medical school a living entity, but was unable to do so on account of the laxity of interest which the students manifested towards this department of knowledge. He established a Museum of Natural History, a chemical laboratory, and a dissecting-room for the University, all of which were in full working order at this time in Leyden. John held the Aberdeen Chair for nine years, when he resigned, and two years afterwards became Professor of Physics in the University of Edinburgh, lecturing alternately with Cullen, becoming sole professor on his death. Dr. James Gregory (tertius), Dr. John's son, was born in 1753, succeeded his father in the professorship, and was the author of the " Conspectus Theoreticæ Medicinæ." It was this last Gregory who met Burns in Edinburgh at Lord

SIR JAMES MCGRIGOR.

(An alumnus of Aberdeen.)

Monoboddo's house, himself an alumnus of Aberdeen University, and who is referred to in the line

Now worthy Gregory's Latin face.

He corresponded with Burns and presented him with an English translation of Cicero's Select Orations, a favour, as Burns described it, " from the truely worthy and learned Dr. Gregory I shall preserve to my latest hour as a mark of the gratitude, esteem, and veneration I bear the donor. So help me God." His two sons, William and Duncan, devoted themselves to chemistry. Aberdeen medicine, no less than Edinburgh, owes much to this distinguished family.

### HOW TO GIVE SUBSISTENCE AND DEGREES.

When Dr. Samuel Johnstone visited Aberdeen the post of "Mediciner" at the University was regarded as a source of "decent subsistence" to a baronet who had no estate to support his title. Dr. William Chalmers (1782) tried to revivify this office by instituting a mild examination of candidates for the M.D. degree. It had previously been given on the recommendation of doctors of reputation. George Cheyne, for instance, was allowed "to be graduate Doctor in medicine because he is not only our own Countryman and at present not rich, but is recommended by the ablest and most learned physicians in Edinburgh as one of the best mathematicians in Europe." From 1792 till 1839 no lectures were delivered on medicine in Aberdeen University, but degrees were given. Not till 1817 was it thought of requiring something of a guarantee that those upon whom the degree was conferred were not utterly illiterate and destitute of some elementary notions of scientific knowledge.

### DISTINGUISHED ABERDEEN DOCTORS.

Among the other eminent men who have thrown distinction on the Aberdeen University may be

DR. NEILL ARNOTT.

mentioned Dr. Thomas Burnett, who was physician to King Charles II., James II., William III., and Queen Anne; Sir William Fordyce and Sir William Farquhar were physicians to George III.; Sir James McGrigor, who was declared by the Duke of Wellington to be one of the most industrious able public servants he had ever met. Within our own memory distinctions have come to Aberdeen University by men who are the exact antitheses of the learned philosophers of medicine, though no less wise and eminent, like Drs. Neil Arnott, Keith, Pirrie, Sir Andrew Clark, and last, but not least, the distinguished and zealous anatomist Sir John Struthers, who, although not educated in Aberdeen University, certainly did much to enhance its reputation and the value of its teaching. His large additions of specimens to the Anatomical Department, both human and comparative, were worthy of the great University.

### FROM MONASTICISM TO SCIENCE: A STRUGGLE AND A MORAL.

In this sketchy summary of the origin and progress of science along the highway of medicine as it relates to Aberdeen University we can only link up the more outstanding features which entitle it to a high rank, not only from its antiquity and the brilliance of its early hopes and aspirations, but by its endurance in spite of obstacles arising from its geographical position, its political environment, its religious antagonisms, and the inherent combativeness which attaches to the northern dispensation, making them slow to adopt new methods. The Aberdeen University has held its way and progressed from a monastic clericalised adjunct of popery to a modern fully equipped high-class centre of medical learning—a credit at once to the town and to the country. It is usual to connect the reputation of a school with the names of the great ones it has given to the world. Aberdeen University has attracted to it some of the greatest men; it has sent out to the nations of the world multitudes of practitioners of medicine who have brought it credit and renown—a reflex of its early life and a prouder record than architectural display. The polarity of the second magnet is opposite to that of the first, and so the induced intellectual activity of the modern University may be different from that which originally set it going; but this short historical *résumé* of its medical work clearly demonstrates a continuity of force and intellect from which the present-day greatness has been derived and upon which it is dependent. The influence of the past has a much more powerful effect on the present than the casual spectator is apt to believe.

Aberdeen University (King's College and Marischal College became part of the same University in 1858) is now converted into a high-class modern institution. That conversion is not due to influences which have suddenly burst over it from extraneous sources, but it has been induced by the forces of the past acting continuously and continuing to act upon the present, although at the outset these forces were totally and widely opposed to each other. The lesson these ancient institutions have to teach us is one of continuous development and progress. And still it is the hope that progress will continue through other centuries yet to come, and that future retrospects will say of Aberdeen what Shelley said of the renowned Italian University to which so many of its teachers and students of medicine had to resort for enlightenment in the early days, and of which Aberdeen was but a replica:

> Padua, in thy halls of learning
> Still the lamp of light is burning.

THE WELCOME.

This was the keynote that inspired with a language dignified by simplicity and sincerity the Lord Chancellor of the University, Lord Strathcona, in extending a welcome on Tuesday, the 25th inst., to the multitude of distinguished guests from all parts of the world who had come to honour the memory of the founders of this notable University and its work. Addressing Sir Frederick Treves, the Lord Rector (who stood on his right), and the Lord Provost of Aberdeen, the members of the University, and all present, he said: "We are here assembled at the last of what has grown to be quite a long series of University celebrations —Edinburgh, Bologna, Dublin, Harvard, Yale, Glasgow, and now our own city of learning, Aberdeen. We look back to the vista of 400 years on work accomplished, and forward to what still remains to be done. It is indeed a pleasant duty to me to thank those who have come here from foreign countries as well as from the homeland to honour Aberdeen with their presence on this occasion.

[By permission of the *British Medical Journal*.

AN OLD SEVENTEENTH CENTURY PRINT OF MARISCHAL COLLEGE, ABERDEEN.

[By permission of the *British Medical Journal*.

MARISCHAL COLLEGE AT THE PRESENT DAY.

This gathering represents a world Parliament, the Universities, the great federators of the modern world. Those studies which they cherish in common throughout the whole civilised world constitute, as a great writer has said, *une grande patrie*, which transcends the boundaries of restricted and too often hostile nationalities, and which is sustained by no war, menaced by no foe. It is in these studies, pursued everywhere in the same spirit, that the best thought and highest intelligence of our times find rest in communion. It is not for me to praise Aberdeen, but she is worthy. In the 400 years that have passed since the first foundation of King's College our University has had a varied history which has been connected with the national life, and has at the same time impressed itself deeply on the country, as intimately whole, but more especially in the north-eastern district of Scotland. In the far-off time when the Papal Bull was issued founding our college the people of the locality are described as rude, ignorant of letters, almost untamed. We have evidence, however, that then, as now, they had that spirit, that quality of positiveness or, let us say, persistent endeavour which has served Scotsmen in good stead throughout past centuries, with a veneration for the traditions handed down to them by their ancestors which may have made them indisposed, without due and deep consideration, to accept a new order of things. To-day there are the hosts of the learned societies of Europe and America, and through these four centuries Aberdeen has kept the sacred torch of learning and scholarship burning brightly."

## Figures and Tables

**Figure f1:**
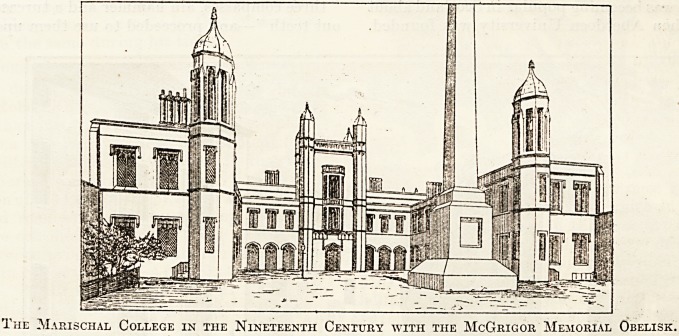


**Figure f2:**
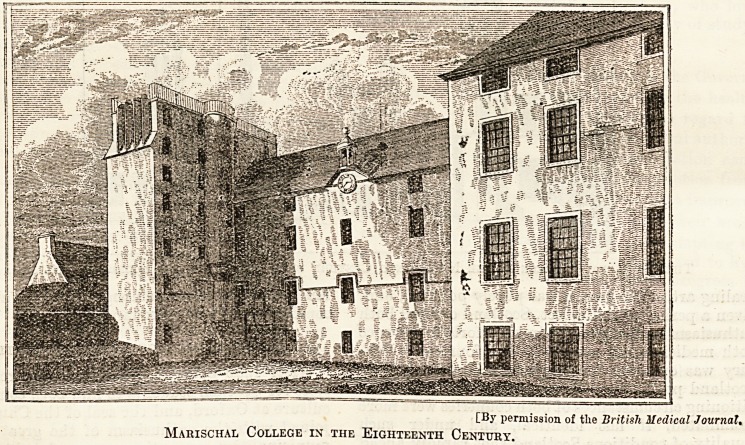


**Figure f3:**
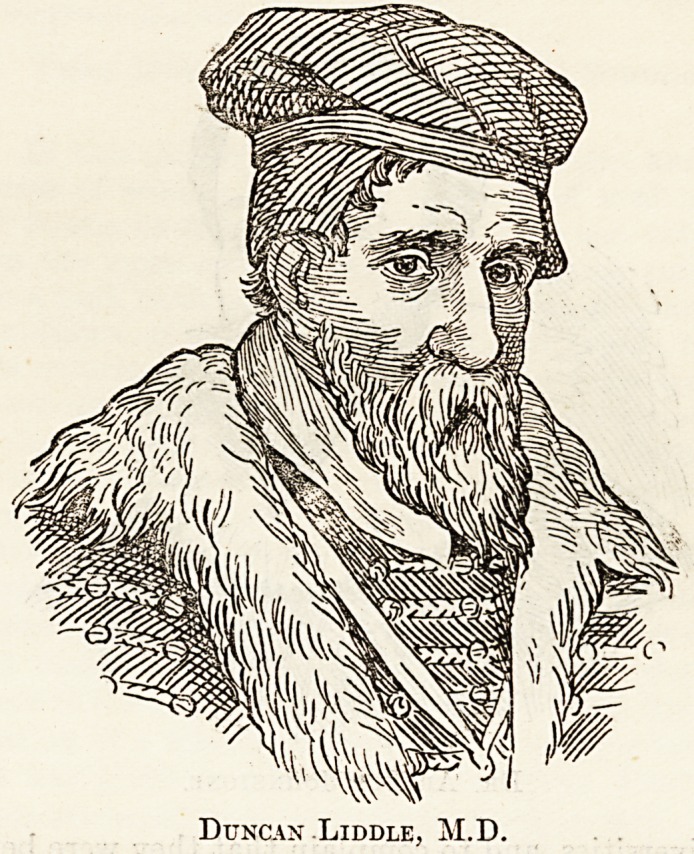


**Figure f4:**
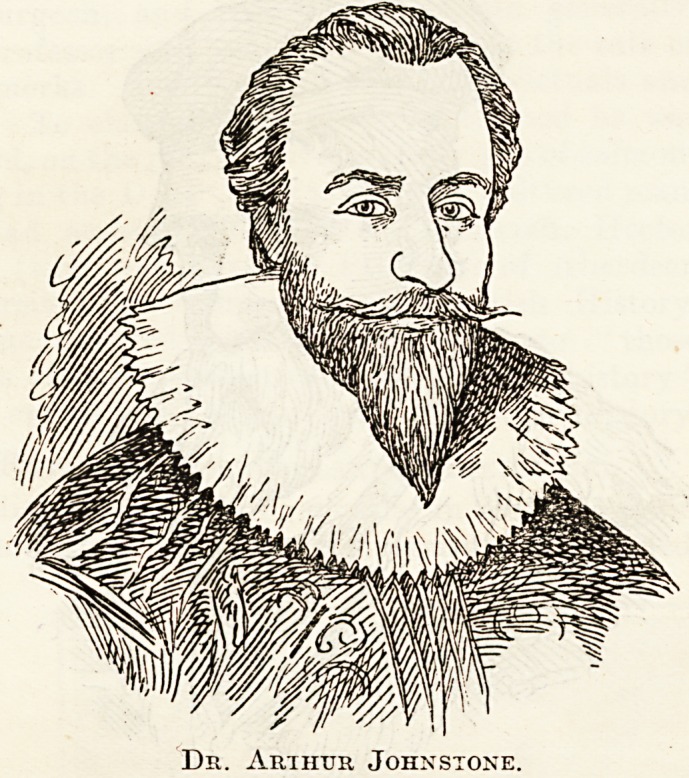


**Figure f5:**
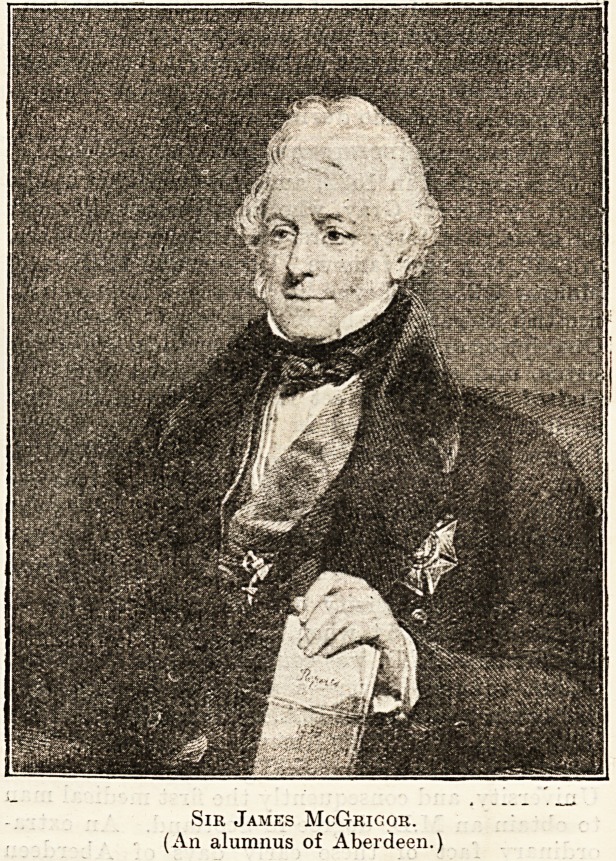


**Figure f6:**
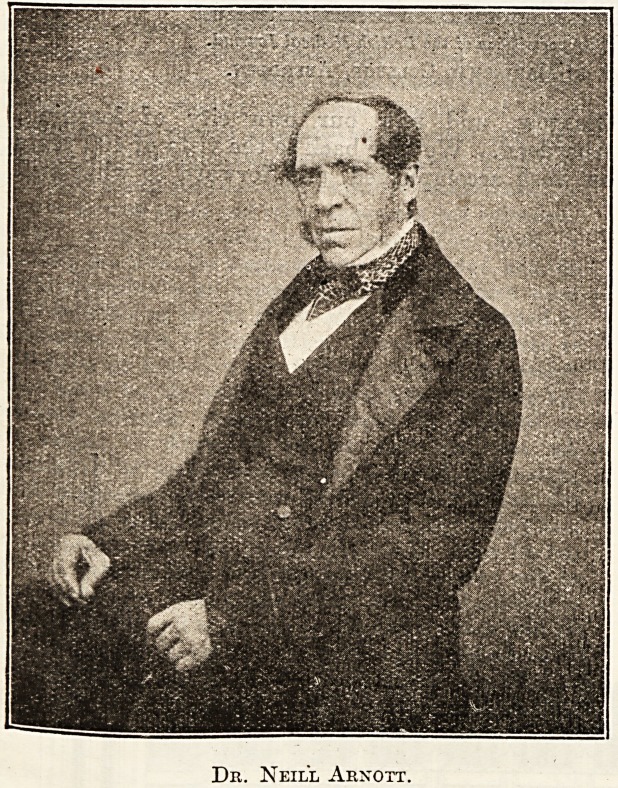


**Figure f7:**
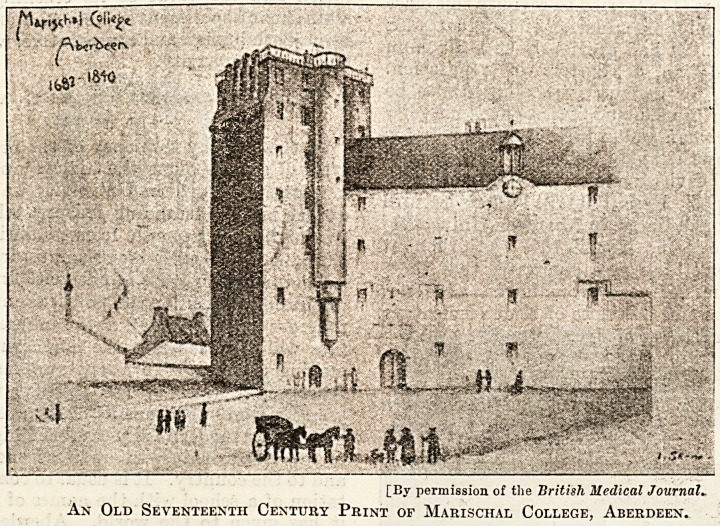


**Figure f8:**